# Submarine Rescue Decompression Procedure from Hyperbaric Exposures up to 6 Bar of Absolute Pressure in Man: Effects on Bubble Formation and Pulmonary Function

**DOI:** 10.1371/journal.pone.0067681

**Published:** 2013-07-02

**Authors:** Jean-Eric Blatteau, Julien Hugon, Olivier Castagna, Cédric Meckler, Nicolas Vallée, Yves Jammes, Michel Hugon, Jan Risberg, Christophe Pény

**Affiliations:** 1 Equipe Résidante de Recherche Subaquatique Opérationnelle (ERRSO), Institut de Recherche Biomédicale des armées (IRBA), Toulon, France; 2 Bf-Systèmes, Technopole de la Mer, La Seyne sur Mer, France; 3 Faculté de Médecine Nord, Unité Mixte de Recherche (UMR-MD2), Marseille, France; 4 Service de Médecine Hyperbare et Expertise Plongée (SMHEP), Hôpital d’Instruction des Armées Sainte-Anne, Toulon, France; 5 Norwegian armed forces, joint Medical Services, Office of submarine and diving medicine, Bergen, Norway; 6 Cellule Plongée Humaine et Interventions Sous la Mer (CEPHISMER), French Navy, Toulon, France; Texas A&M University-Corpus Christi, United States of America

## Abstract

Recent advances in submarine rescue systems have allowed a transfer under pressure of crew members being rescued from a disabled submarine. The choice of a safe decompression procedure for pressurised rescuees has been previously discussed, but no schedule has been validated when the internal submarine pressure is significantly increased i.e. exceeding 2.8 bar absolute pressure. This study tested a saturation decompression procedure from hyperbaric exposures up to 6 bar, the maximum operating pressure of the NATO submarine rescue system. The objective was to investigate the incidence of decompression sickness (DCS) and clinical and spirometric indices of pulmonary oxygen toxicity. Two groups were exposed to a Nitrogen-Oxygen atmosphere (pO_2_ = 0.5 bar) at either 5 bar (N = 14) or 6 bar (N = 12) for 12 h followed by 56 h 40 min resp. 60 h of decompression. When chamber pressure reached 2.5 bar, the subjects breathed oxygen intermittently, otherwise compressed air. Repeated clinical examinations, ultrasound monitoring of venous gas embolism and spirometry were performed during decompression. During exposures to 5 bar, 3 subjects had minor subjective symptoms i.e. sensation of joint discomfort, regressing spontaneously, and after surfacing 2 subjects also experienced joint discomfort disappearing without treatment. Only 3 subjects had detectable intravascular bubbles during decompression (low grades). No bubbles were detected after surfacing. About 40% of subjects felt chest tightness when inspiring deeply during the initial phase of decompression. Precordial burning sensations were reported during oxygen periods. During decompression, vital capacity decreased by about 8% and forced expiratory flow rates decreased significantly. After surfacing, changes in the peripheral airways were still noticed; Lung Diffusion for carbon monoxide was slightly reduced by 1% while vital capacity was normalized. The procedure did not result in serious symptoms of DCS or pulmonary oxygen toxicity and may be considered for use when the internal submarine pressure is significantly increased.

## Background

When a disabled submarine is unable to surface, survivors awaiting rescue may be exposed to raised internal submarine pressure. Such an increased pressure may result from a variety of causes including flooding of compartments or release of gas from ruptured air banks. Previous incidents suggest that the time spent at elevated pressure, while waiting for rescue, may be long enough for the crew to be considered “saturated”, i.e. all body tissues has reached equilibrium with ambient inert gas (usually Nitrogen). A direct ascent to the surface when saturated carries a high risk of decompression sickness (DCS) and death, as demonstrated by the accident of the submarine Pacocha [1]. Well documented human data on shallow air saturation exposures suggest that direct ascent is not associated with DCS symptoms when the subject is saturated at a pressure less than 1.8 bar of absolute pressure [2]. The ambient pressure is the primary predictor of DCS incidence and death following ascent to surface after saturation exposure. Neurological DCS symptoms predominate after exposure to lower pressure (but more than 1.8 bar) [2]. As pressure increases, there is a gradual shift towards the more severe cardiopulmonary DCS that rapidly becomes fatal [3].

**Figure 1:**
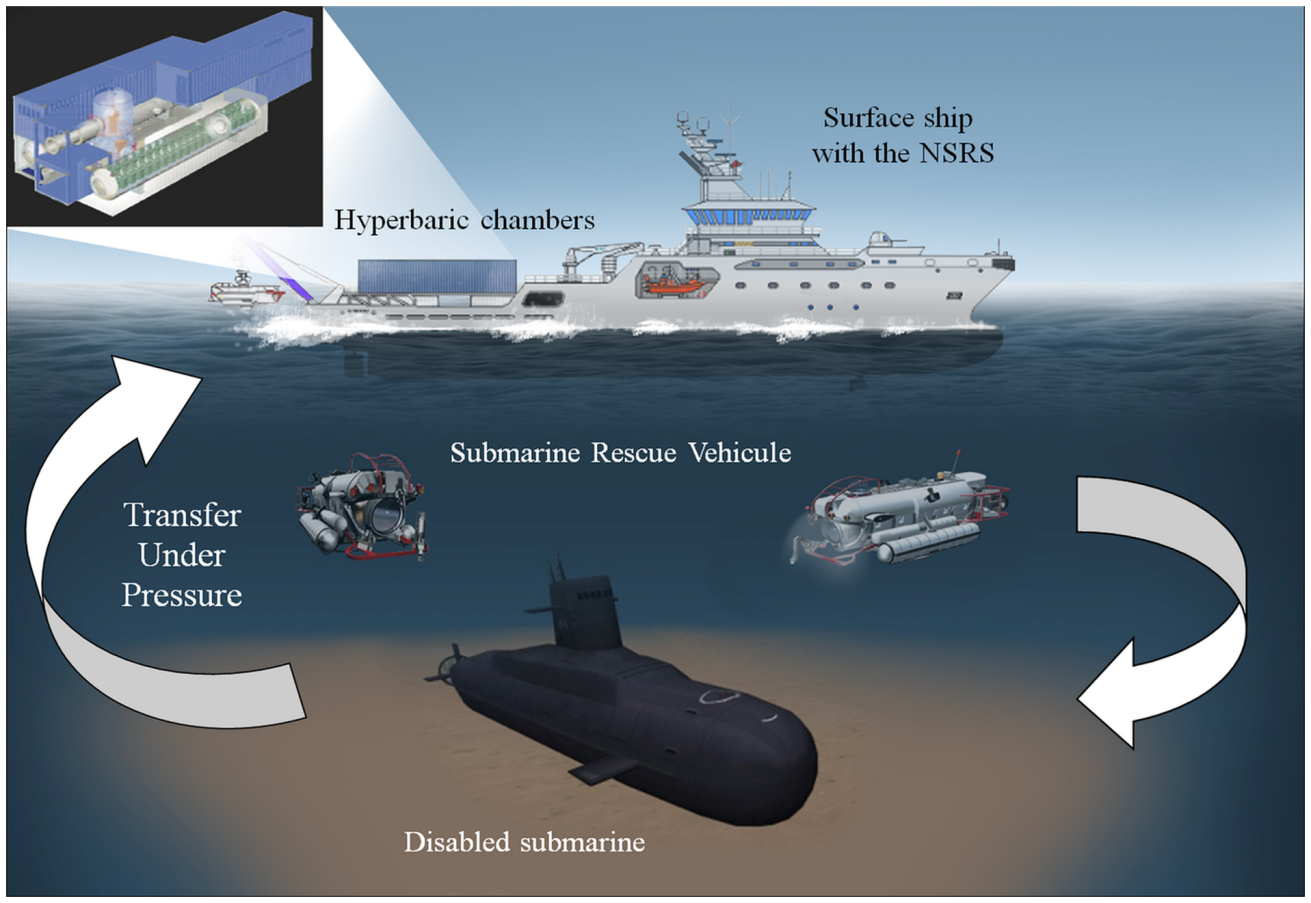
The NATO submarine rescue system (NSRS), using a submarine rescue vehicle, allows the transfer under pressure of pressurised crew members from a disabled submarine to hyperbaric chambers on a surface ship.

Following a rescue from a disabled submarine, the crew members would require controlled decompression, as they may be at risk of severe DCS if decompressed immediately to surface pressure. The NATO Submarine Rescue System (NSRS) is a submarine rescue system which has the capability of transferring submariners from a submarine, via a submarine rescue vehicle (SRV) to a deck hyperbaric chamber without alteration in ambient pressure. The submariners are then subjected to a controlled and final decompression on a surface ship (Figure 1). The SRV is able to dive down to 610 meters of sea water (msw), locate and attach itself to the distressed submarine. Following pressure equalisation, up to 15 rescuees can be transferred into the SRV, before returning to the surface. Once recovered to the surface ship, the SRV is connected to the hyperbaric chamber allowing the transfer and safe decompression of pressurised crew members (capacity of 72 subjects), whilst the SRV performs further recovery dives [4]. The system is stipulated to be able to accept the first rescues within 72 h from start of mobilisation.

The issue of safe and efficient decompression of submariners being rescued from a disabled submarine has been discussed for a long time. Recent advances in submarine rescue systems with NSRS have allowed the transfer under pressure and called for proper procedures to avoid DCS.

The choice of a decompression schedule for such rescuees depends on a variety of factors. The pressure in the disabled submarine escape compartment to which the crew members have been exposed is the main factor. It is believed that the likelihood to survive for 72 h or more in a disabled submarine is reduced when the internal pressure is more than 6 bar of absolute pressure [5]. In circumstances where there is little or no constraint on decompression time, the optimal decompression procedure is to use a slow decompression rate, breathing air with some periods of pure oxygen breathing to shorten decompression time [5]. This provides the safest decompression but will delay evacuation.

In circumstances where there are extreme constraints on decompression time it may be necessary to utilise schedules with multiple periods of oxygen breathing. Since inhalation of O_2_ for a long period can cause pulmonary oxygen toxicity (POT), these schedules are of considerably shorter durations but carry risks of higher incidence of DCS than in the case of the long air schedules [6]. Moreover, for deeper exposures, when pressure is more than 2.8 bar, the risk of severe POT prohibits the use of oxygen breathing to accelerate decompression. Due to this toxic effect of oxygen on lungs, little experimental work is available to support a proper procedure for air/oxygen saturation decompression deeper than 2.8 bar.

Submariners isolated in a distressed submarine at sea bottom face a significant risk of injuries and illnesses. Being rescued by a SRV to surface will increase the chance of survival and health protection compared to risks associated with escape through the water column to the surface. The health risks associated with the following decompression in the deck decompression chamber, mainly those of decompression sickness and pulmonary oxygen toxicity, are generally considered minor compared to the catastrophic alternative of remaining in the submarine or ascending from depths expected to be lethal. Risk acceptance for side effects and complications of saturation decompression procedures for submariners in this situation is conceived to be higher than for e.g. occupational divers. However, the presence of divers and nurses with experience in hyperbaric medicine are required within the decompression chamber to support the submariners. These chamber attendants are healthy military personnel, and measures should be taken to protect them from illness and injuries while supporting the submariners.

The aim of this study was to evaluate a new saturation decompression procedure. The procedure was designed to decompress submariners exposed to compressed air at 6 bar equivalent to 50 msw which would constitute the maximum permissible internal pressure of the NSRS. The objective was to verify the safety of the procedure for NSRS attendants with respect to decompression sickness and pulmonary oxygen toxicity. We therefore performed repeated clinical examinations, investigated the presence of vascular bubbles and studied lung mechanics using spirometry during decompression from saturation exposures to 40 and 50 msw in 26 subjects. The pulmonary function study was completed before and after the dives by measuring all the lung volumes and pulmonary mechanics using a whole-body pressure displacement plethysmograh. We also used a mathematical model of decompression to simulate and predict bubble formation in different configurations carried to extremes that could not be tested in this study.

## Materials and Methods

### Subjects

Twenty-four medically fit military subjects with a diving experience of 9.7±4.4 yr (mean ± S.D) gave their written consent; procedures conformed to the declaration of Helsinki. The experimental protocol was approved by both the scientific committee for the protection of human subjects (CPP Sud Mediterranée I, ref 1104) and the French national treatment agency (AFSSAPS, ref B111347-70). All the subjects were trained military divers, hyperbaric nurses or diving medical officers, none of them had experienced DCS in the past. Their age was 37.5±5.3 yr with a body mass index of 25.2±1.7 kg m^−2^. Three hyperbaric exposures to 5 bar (40 msw) were carried out in 2011, involving 14 subjects (5 subjects in May and June, 4 subjects in December 2011). Three hyperbaric exposures to 6 bar (50 msw) were carried out in 2012, involving 12 divers (4 subjects in February, March and April 2012). Two subjects who completed exposures to 40 msw were also included in exposures to 50 msw.

### Facility

All saturation exposures were performed in the main hyperbaric chamber of the French navy hyberbaric center located at CEPHISMER (Toulon, France). The pressure chamber complex held three compartments. The system was controlled and monitored from a nearby control console. The console maintained ambient pressure, continuously monitored CO_2_ and O_2_ partial pressures, and allowed adjustment of the decompression rate as needed. Temperature was adjusted to subject comfort and ranged 24–28°C; the relative humidity was 45–60%. When not breathing chamber gas, the subjects breathed Oxygen or Nitrogen-Oxygen gas mixtures as detailed below through tight fitting oro-nasal masks from the built-in breathing system (BIBS).

### Exposure at Depth

To facilitate operational use of the results in the present study, we will report ambient pressure in msw and bar; and partial pressure in bar, recognizing that 10 msw = 1 bar = 101 kPa.

The hyperbaric chambers were compressed with air until 13.8 msw to obtain a pO_2_ of 0.5 bar. Pure nitrogen was then added until the maximum pressure was reached. The divers kept their mask breathing air from surface to 25 msw, and a Nitrox (O_2_-N_2_) mixture from 25 msw until the maximum depth. After homogenization at depth, the subjects were authorized to remove their mask. The compression rate was 0.5 bar min^−1^ (5 msw min^−1^).

We chose an exposure time of 12 h to the maximum pressure, which corresponds to the maximum allowed duration for an intervention of attendants in the NSRS. To avoid the occurrence of POT, the subjects breathed a Nitrox atmosphere with a pO_2_ maintained at 0.5 bar. The decompression schedule for such a Nitrox atmosphere should be adjusted for a higher Nitrogen partial pressure due to the increased fractional content of Nitrogen compared to air. The conventional method for such an adjustment is to calculate the pressure at which compressed air would have the same Nitrogen partial pressure as the Nitrox mixture – “the equivalent air depth”. A Nitrox mixture with raised Nitrogen content would thus require decompression according to an increased equivalent air depth.

Two schedules were tested: 1) 40 msw using Nitrox mixture 10% O_2_–90% N_2_ (equivalent air depth of 46.9 msw) and 2) 50 msw using Nitrox mixture 8.33% O_2_–91.67% N_2_ (equivalent air depth of 59.6 msw).

### Decompression Procedure

The schedule was based on the experience from the “AIRSAT 4” exposure, using slow decompression rates, which were believed to be safe as the subjects developed very few signs of DCS. However significant symptoms of POT related to 48-h exposure in air atmosphere to 5.02 bar (40.2 msw) [7], [8]. A perfusion limited gas model with a controlling compartment of 480 min half-time was used for the calculation of the schedule. Oxygen exposure was assessed by the “Oxygen Toxicity Unit” (OTU) and “Repex” procedure, assuming that 850 OTU was a tolerable cumulative dose for 24 h, 1400 OTU for 48 h and 1860 OTU for 72 h [9]. The OTUs attained during periods of oxygen breathing were added during decompression to control for the permissible thresholds.

#### Decompression from 40 msw

**Figure 2:**
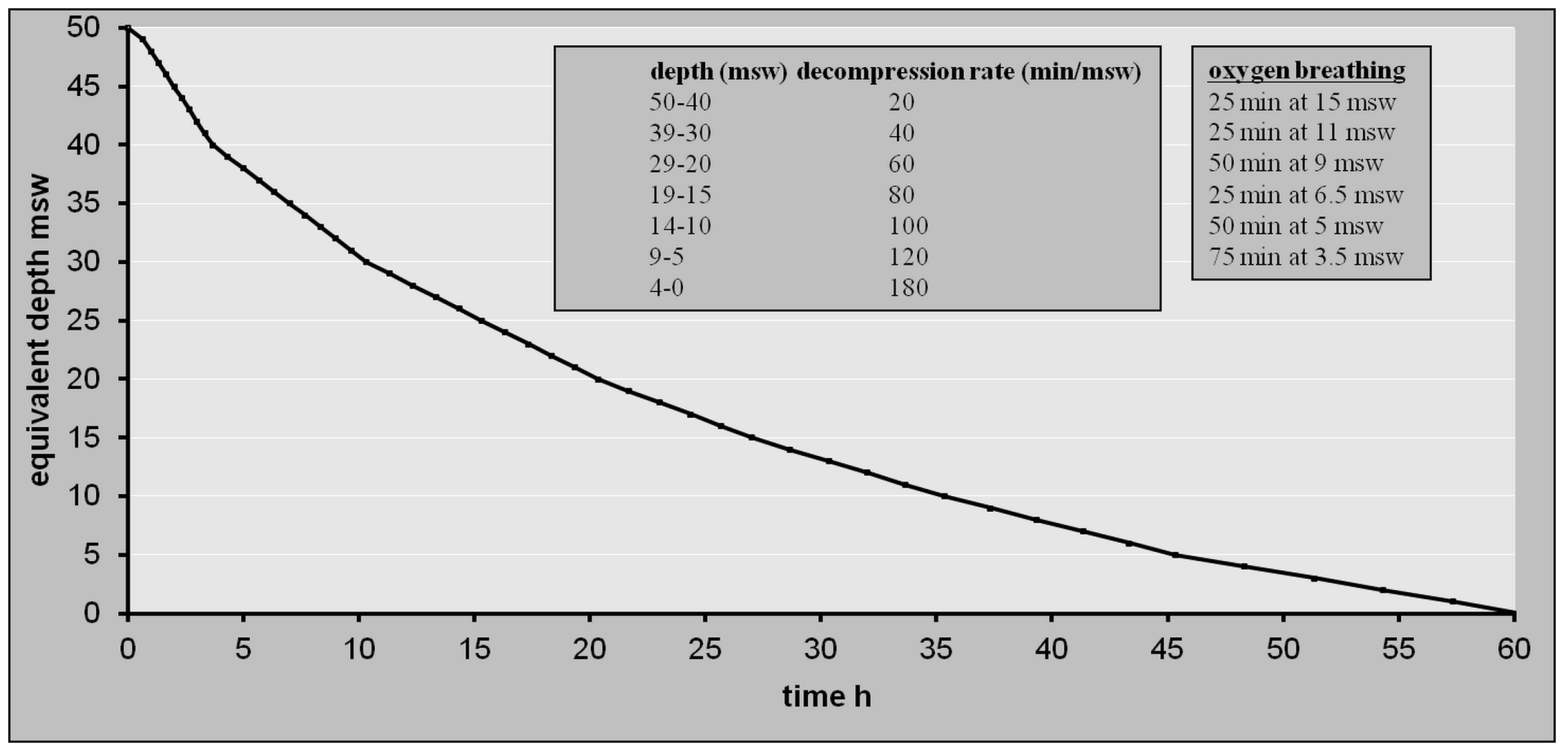
Decompression procedure after saturation exposure to 50 msw. Five min of air breathing (air breaks) were given after 25 min of Oxygen breathing.

After 12 h of exposure to 40 msw, the atmosphere was isobarically switched to air and the decompression initiated simultaneously. Air was maintained as the chamber gas during the succeeding decompression. Decompression rates varied from 40 to 180 min msw^−1^ according to the depth (Figure 2). The chamber reached the surface on day 4 for a total decompression time of 56 h 40 min.

Periods of oxygen breathing were incorporated during the end of decompression:

- in May and June 2011 (n = 10), 4 O_2_ periods were added: 60 min from 12.5 msw, 50 min from 9.5 msw, 75 min from 6.5 msw, and 100 min from 3.5 msw, with a cumulative oxygen dose of 1341 OTU at completion.
- in October 2011 (n = 4), 6 O_2_ periods were added: 30 min from 15 msw, 50 min from 10 msw, 50 min from 8.5 msw, 50 min from 5 msw, 50 min from 4.5 msw and 75 min from 3.5 msw, with a cumulative oxygen dose of 1384 OTU at completion.

Air breaks were added to these periods, using a sequential mode as follows: 30 min O_2_–5 min air −30 min O_2_ or 25 min O_2_–5 min air –25 min O_2_.

#### Decompression from 50 msw

After 12 h of exposure at 50 msw, the atmosphere was isobarically switched to air and the decompression initiated simultaneously. Air was maintained as the chamber gas during decompression. Decompression rate was reduced stepwise from 20 to 180 min msw^−1^ as illustrated in Figure 2. Decompression to surface pressure was reached after a total decompression time of 60 h. The schedule included 6 periods of oxygen breathing during the end of decompression i.e. 25 min from 15 msw, 25 min from 11 msw, 50 min from 9 msw, 25 min from 50 msw, 50 min from 5 msw and 75 min from 3.5 msw. Air breaks were added as follows: 25 min O_2_–5 min air –25 min O_2_. The cumulative dose of oxygen was 1513 OTU at completion.

### Clinical Evaluation

Assessment of decompression was gauged primarily by reported symptomatology related to POT or DCS. DCS symptoms were divided into conventional categories: pain only symptoms (type I) and multisystem or serious symptoms (type II). Pulmonary symptoms including chest pain, cough, chest tightness, dyspnea were also recorded. Divers were required to record symptoms at least every 6 h on prepared questionnaires. Symptoms of inert gas toxicity (Nitrogen narcosis) were also noted and specific tests for narcosis evaluation were performed; these results were published separately [10].

In addition, a diving medical officer interviewed and physically examined each diver twice daily throughout the exposure and recovery periods. The diving medical officer was also responsible for ultrasound monitoring and spirometry.

### Bubble Detection

Venous microemboli were investigated by means of a pulsed Doppler ultrasound apparatus equipped with a 2 MHz probe (BF Systemes company, France) directed to the right cardiac cavities. The system has been validated for hyperbaric use. The signals were listened to and interpreted in real time by the diving medical officers and saved for additional analysis. Precordial monitoring was performed in supine position for 3 min at rest and after two lower limb flexions. The bubble count was graded according to the Spencer scale [11]. Depending on the depth, 2–4 detections were performed daily during the decompression phase. Bubble detection was monitored constantly during the first hour after surfacing.

### Bubble Simulation

In our study, we did not evaluate the effects of decompression after a prolonged exposure (e.g. 48 h or more) allowing complete gas equilibration (saturation), but rather a 12 h exposure expected to saturate most, but not all, compartments (sub-saturation). However, we used a mathematical model (BORA model, BF systemes) to simulate and predict bubble formation for extended exposure [12], [13]. Under conditions of saturation diving, compartments controlling decompression rate are very slow in terms of gas exchange. The compartments retained in the model after calibration have gas half-saturation time (T_1/2_) ranging from 380 min to 420 min. The BORA model was calibrated with data from previous studies, using vascular microbubble scores (Ultrasound Doppler) after air or Nitrox saturation [7], [14]. Bubble simulation was obtained by considering an average response from a conceptual “central tissue” with T_1/2_ = 400 min and the total bubble volume (v_b_, ml_gas_ ml_tissue_^−1^) of all microbubbles formed in this tissue. A correspondence between the total bubble volume v_b_ and bubble grade according to the Spencer scale (from 0 to IV) was calculated i.e. grade 0 for v_b_<0.02, grade I for v_b_ [0.02–0.04], grade II for v_b_ [0.04–0.07], grade III for v_b_ [0.07–0.12] and grade IV for v_b_≥0.12 ml_gas_ ml_tissue_^−1^.

### Spirometry

Spirometry was performed before, during and after the exposures. All spirometry was carried out with the subject standing up. Before and after the dives, a whole body pressure displacement plethysmograph (Masterlab, Jaeger, The Netherlands) allowed us to measure the forced vital capacity (FVC), total lung capacity (TLC), residual volume (RV), and the RV/TLC ratio. The Masterlab device also allowed measurement of the pulmonary diffusing capacity for carbon monoxide (DL_CO_) and the DL_CO_ was adjusted to the alveolar volume (DL_CO_/VA). During the dives and also for the pre- and post-dive evaluations, electronic spirometers (Spirobank II, MIR, Waukesha, USA) were used to measure the FVC, forced expired volume in 1s (FEV_1_), average forced expiratory flow from 25 to 75% of expired volume (FEF_25–75_) and peak expiratory flow rate (PEFR). The tests were repeated at least once on each session with the best effort being retained. The results are presented in units of body temperature, surface pressure and saturated gas (BTPS).

During hyperbaric exposure, we used the Spirobank II hand-held spirometer, previously tested [15] and calibrated for hyperbaric use. Measurements were performed immediately before compression, at maximal pressure, and daily during the decompression. The last measurement was carried out just after surfacing. Pre-exposure measurements were completed 3 days before, while and post-exposure measurements were performed 6 h after the experiment in the respiratory department of Ste Anne military hospital (Toulon, France).

### Statistics

Sigmastat 3.0 software program (SPSS inc., Chicago, Illinois) was used for statistical analysis. Data, presented as median ± interquartile range, were analysed using non-parametric statistics because of the small sample size. Comparisons for the repetitive data were analyzed across time with a repeated measure analysis on ranks and Dunn’s test for post hoc analysis. Differences between two groups were analysed by a Mann-Whitney test, whereas matched comparisons within groups used a Wilcoxon test. A difference was considered as statistical significant for p-values<0.05.

## Results

### Clinical Symptoms

Chest tightness during deep inspiration was commonly reported during the initial phase of decompression from 30 to 20 msw in both groups (42 and 43% for subjects exposed to 40 and 50 msw respectively). Precordial burning sensations were also noted in 42% (resp. 25%) of subjects during the last periods of oxygen breathing in the final phase of decompression after exposures to 40 msw (resp. 50 msw).

Three subjects (12%) had minor symptoms during exposures to 40 msw. Subjects described from 20 msw sensations of heaviness in the joints i.e. the two shoulders (2 subjects) and one elbow (1 subject), regressing spontaneously during decompression from 10 msw. After surfacing (from exposures to 40 msw), 2 subjects (8%) also experienced joint discomfort disappearing gradually, without therapeutic intervention. Four hours after surfacing, one of these subjects presented a feeling of heaviness in the shoulder that disappeared after 90 min. The other described the recurrence of mild discomfort in the elbow after surfacing, persisting for several days, then disappearing spontaneously. Examinations including bone scintigraphy and MRI were performed for these two subjects. No periarticular or bone involvement was found. Moreover, none of these symptomatic subjects showed detectable circulating bubbles during or after exposures.

70% of subjects from exposures to 40 msw reported a state of tiredness persisting for 24 to 72 h after surfacing.

During and after exposures to 50 msw, no subjects report joint symptoms or residual fatigue.

### Bubble Detection

The presence of circulating bubbles was observed in 3 subjects (12%) with only low bubble grades (Spencer grade I). The bubbles were recorded 4 times, at 20 msw for 2 subjects (exposures to 50 msw), and ∼9 msw for 2 subjects (from exposures to 40 and 50 msw). One of these subjects, exposed to 50 msw, had bubbles both at 20 and 9 msw. No circulating bubbles were detected after surfacing.

### Bubble Simulation

**Figure 3:**
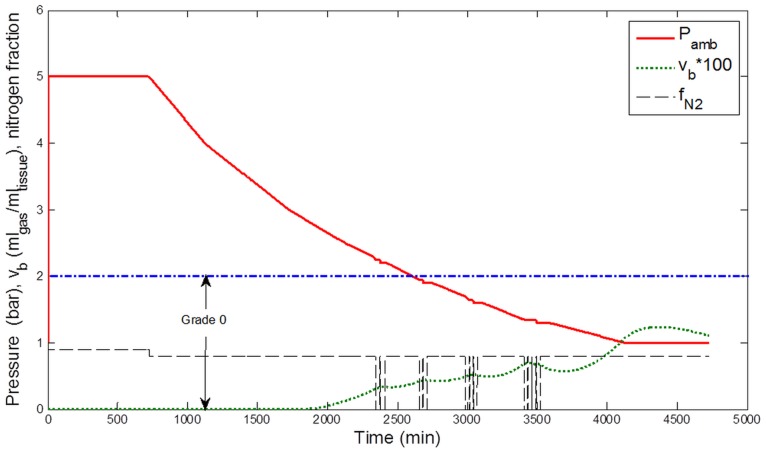
Simulation results for the decompression profile from 12-h exposure to 5 bar (40 msw). Ambient pressure (Pamb, bar), breathing gas Nitrogen fraction (f_N2_), and estimated total bubble volume (v_b_, ml gas ml tissue^−1^) in a conceptual tissue compartment (T_1/2_ = 400 min). The corresponding estimated bubble grade according to the Spencer scale is indicated. Periods of pure O_2_ breathing appear shaded in the figure.

**Figure 4:**
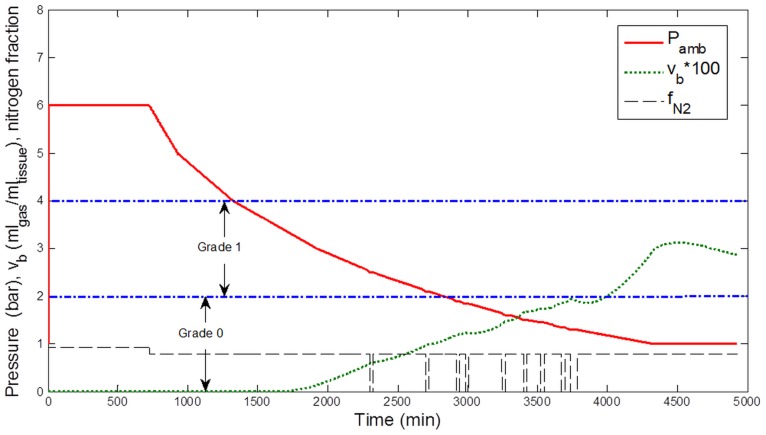
Simulation results for the decompression profile from 12-h exposure to 6 bar (50 msw). Ambient pressure (Pamb, bar), breathing gas Nitrogen fraction (f_N2_), and estimated total bubble volume (v_b_, ml gas ml tissue^−1^) in a conceptual tissue compartment (T_1/2_ = 400 min). The corresponding estimated bubble grade according to the Spencer scale is indicated. Periods of pure O_2_ breathing appear shaded in the figure.

**Figure 5:**
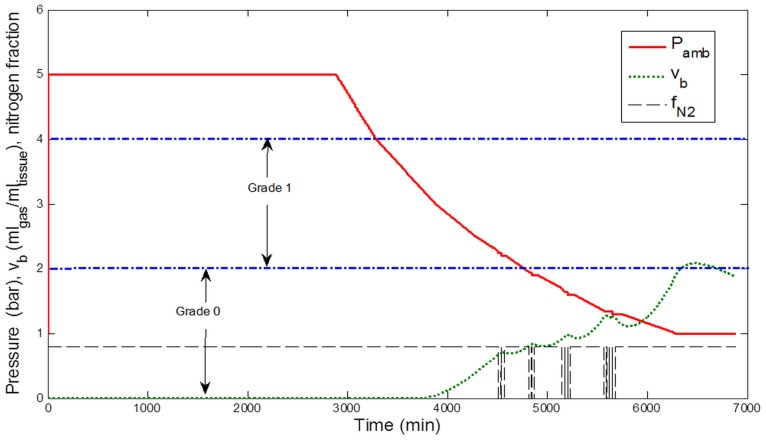
Simulation results for the decompression profile from 48-h air exposure to 5 bar (40 msw). Ambient pressure (Pamb, bar), breathing gas Nitrogen fraction (f_N2_), and estimated total bubble volume (v_b_, ml gas ml tissue^−1^) in a conceptual tissue compartment (T_1/2_ = 400 min). The corresponding estimated bubble grade according to the Spencer scale is indicated. Periods of pure O_2_ breathing appear shaded in the figure.

**Figure 6:**
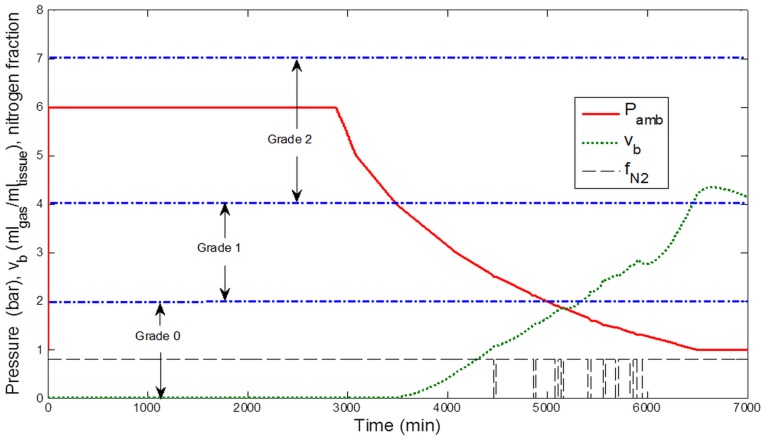
Simulation results for the decompression profile from 48-h air exposure to 6 bar (50 msw). Ambient pressure (Pamb, bar), breathing gas Nitrogen fraction (f_N2_), and estimated total bubble volume (v_b_, ml gas ml tissue^−1^) in a conceptual tissue compartment (T_1/2_ = 400 min). The corresponding estimated bubble grade according to the Spencer scale is indicated. Periods of pure O_2_ breathing appear shaded in the figure.

Figures 3–6 display the results of simulations conducted with the calibrated model, assuming that the circulating bubble flow reflected at any time the total volume of bubbles v_b_ (ml_gas_ ml_tissue_^−1^) formed in a conceptual central compartment.

According to the correspondence between v_b_ and the Spencer scale, a bubble grade 0 for the exposures to 40 msw and a grade I for the exposures to 50 msw were predicted, consistent with Doppler measurements (Figures 3 and 4).

Two other configurations were also studied: 48-h exposures in air atmosphere to 40 msw and 50 msw (Figures 5 and 6). After simulation, the model predicted a bubble grade I for the exposures to 40 msw and a grade II for the exposures to 50 msw.

Table 1 summarizes the bubble grades resulting from different simulated configurations and displays the range of bubble grades most likely to be observed. The table shows that oxygen breathing would tend to predict roughly a halving of estimated bubble generation.

**Table 1:** Averaged bubble grades predicted from mathematical simulation in different configurations of exposures, with or without periods of pure oxygen breathing.

depth	duration	gas	O_2_periods	Vb _(mlgas mltissue-1)_	bubblegrades
40 msw	12 h	Nitrox	yes	0.015	0–I
50 msw	12 h	Nitrox	yes	0.03	0–I
40 msw	12 h	Nitrox	no	0.045	I–II
50 msw	12 h	Nitrox	no	0.065	II–III
40 msw	48 h	Air	yes	0.02	0–I
50 msw	48 h	Air	yes	0.045	I–II
40 msw	48 h	Air	no	0.055	II
50 msw	48 h	Air	no	0.075	II–III

v_b_ is the total bubble volume in a conceptual tissue compartment (T_1/2_ = 400 min).

### Pulmonary Function

#### Measurements during hyperbaric exposures (Figures 7–10)

During the two exposures to 40 and 50 msw, we found a significant reduction in expiratory flows by 48–53% for PEFR, 24–32% for FEV_1_, 23–25% for FEV_1_/FVC, and 47–49% for FEF_25–75_. Recovery was gradual during decompression, except for FEF_25–75_ which remained significantly decreased by 48–49% at 25 msw, before progressive recovery.

**Figure 7:**
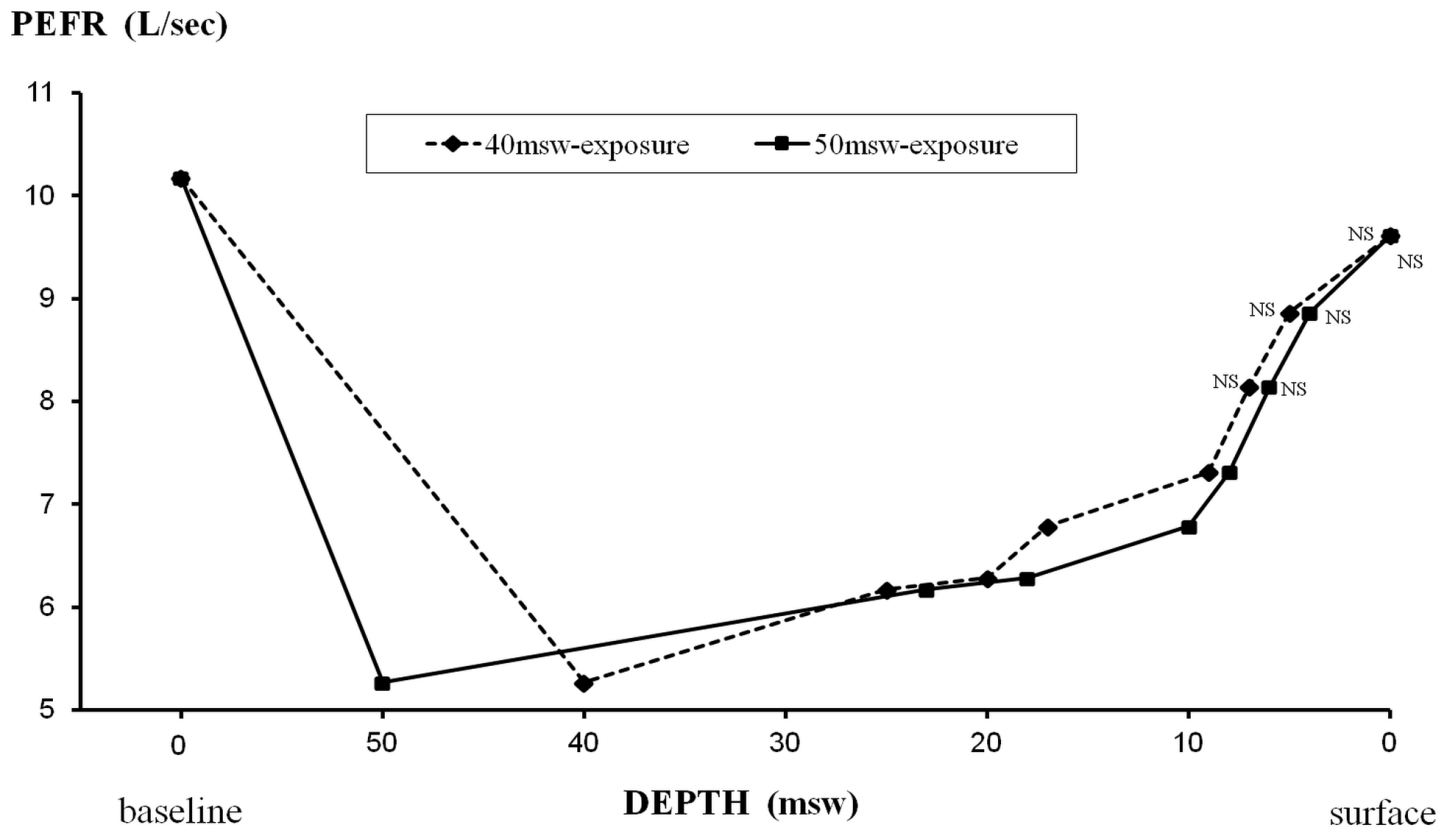
Changes in PEFR during exposures to 40 msw (dashed line) and 50 msw (solid line). Statistically significant decrements from pre exposure values were found except for values indicated as non significant (NS).

**Figure 8:**
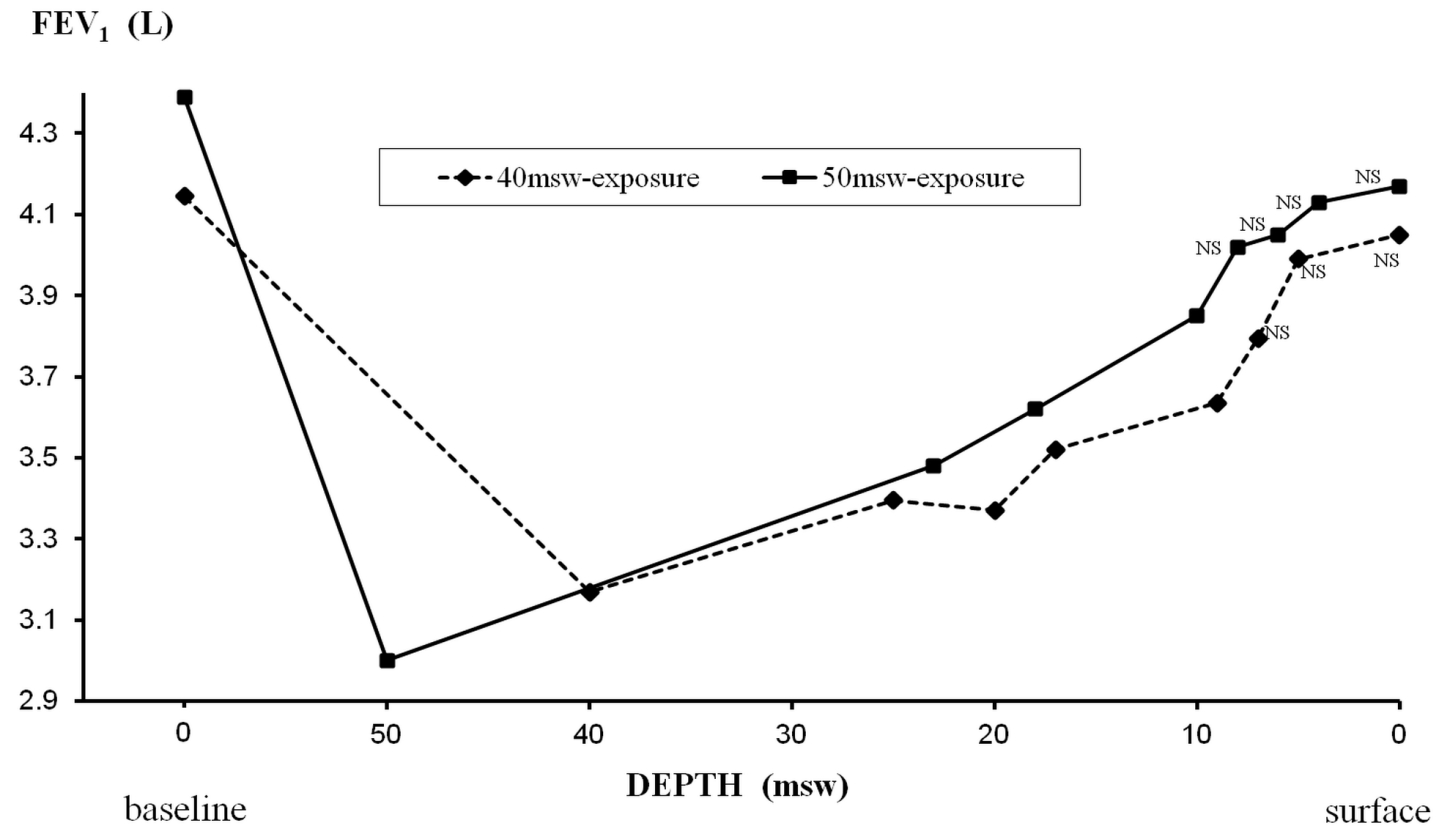
Changes in FEV_1_ during exposures to 40 msw (dashed line) and 50 msw (solid line). Statistically significant decrements from pre exposure values were found except for values indicated as non significant (NS).

**Figure 9:**
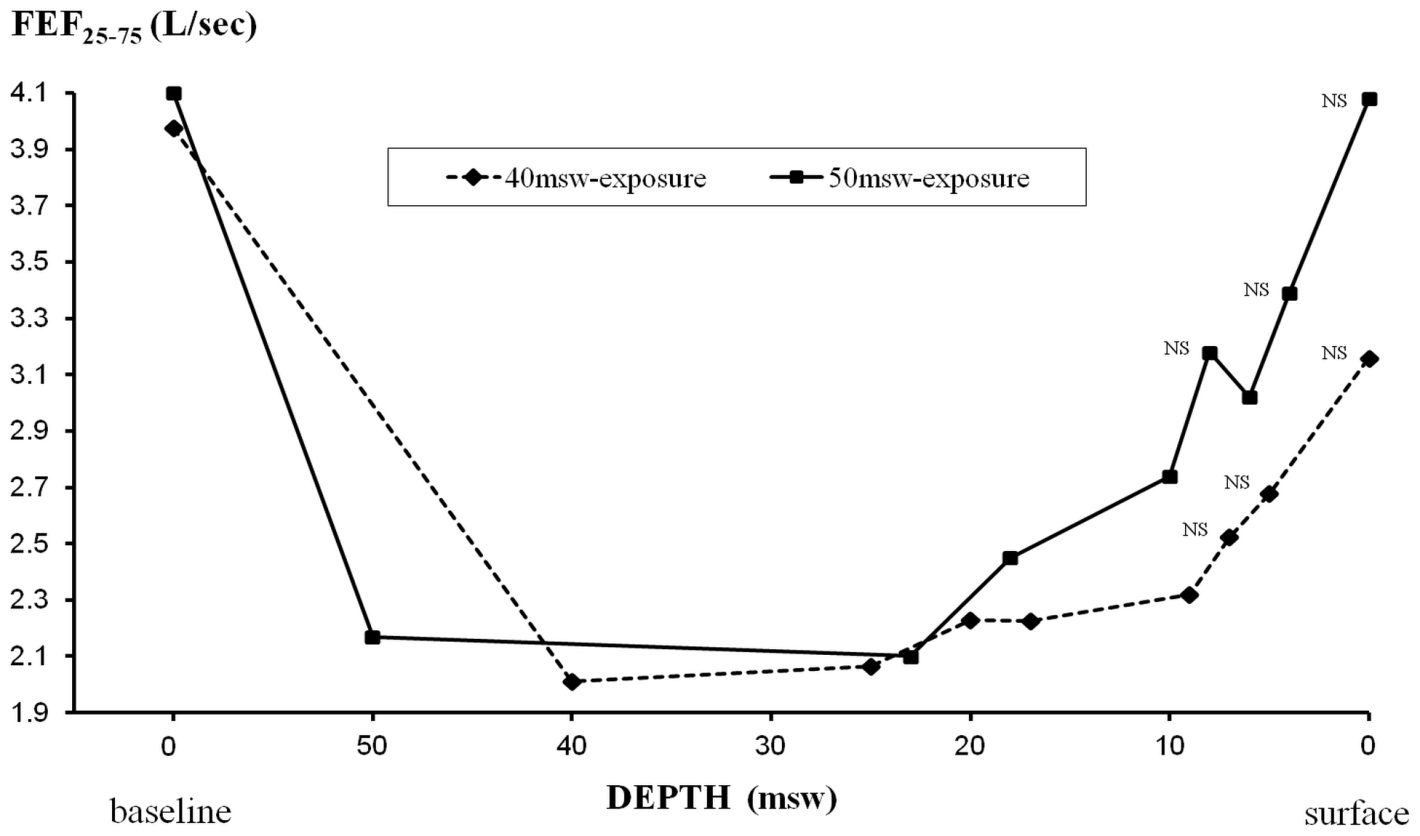
Changes in FEF_25–75_ during exposures to 40 msw (dashed line) and 50 msw (solid line). Statistically significant decrements from pre exposure values were found except for values indicated as non significant (NS).

There was also a significant decrease in FVC observed from 40 to 20 msw with a maximum (−8%) at 25 msw in the 40-msw exposures. The reduction of FVC during decompression in the 50-msw group was quite comparable (−9% at 25 msw), but did not reach statistical significance.

#### Pre/post measurements (Table 2)

FEF_25–75_ decreased by 18–21% in both groups. FEV1 decreased by 5% and the FEV1/FVC ratio decreased from 78 to 76% in the 50-msw group, though no statistical changes in these parameters were observed in the 40-msw group. FVC remained unchanged in both groups, while we noted a slight but significant decrease by 0.9% in DL_CO_ only for the exposures to 40 msw.

At discharge, all spirometric indices were unchanged compared to pre-exposure values.

## Discussion

### Symptoms and Findings Related to Decompression Stress

No case of objective DCS symptom was observed, however some subjects experienced minor subjective joint symptoms, disappearing without treatment. Such minor and transient signs indicate a mild degree of decompression stress and can be interpreted as type 1 DCS symptoms [16]. However, since no subjects showed objective or persistent DCS symptoms requiring therapeutic intervention, we consider the tested procedure to be sufficiently safe for NSRS attendants to be submitted to a similar hyperbaric exposure.

One possible explanation for the presence of circulating bubbles detected early from 20 msw during decompression (50-msw group) could be the relatively fast initial decompression rate (20 min msw^−1^ from 50 to 40 msw and 40 min msw^−1^ from 40 to 30 msw). The use of a period of oxygen breathing from 15 msw appeared therefore fully justified to limit this initial phase of bubble formation. Interestingly, we can note that 50-msw exposures did not result in joint symptoms or fatigue. We speculate that this might be due to a better distribution of O_2_ periods, starting earlier during decompression.

The supersaturation during the initial part of decompression was significantly higher in both groups in the present study than the situation would have been with NSRS chamber attendants exposed to compressed air at the same ambient pressure. Moreover, since NSRS attendants will have to work in a compressed air atmosphere, their exposure to the maximum pressure should be limited to avoid POT. Due to a shorter exposure time and lower pN_2_ compared to the present study, the risk of DCS should be lower than in this study.

In the present study, the risk of DCS to crew members from a disabled submarine exposed to days of raised ambient pressure, has not been investigated. However, to assess this risk for the crew members subjected to such a state, we estimated the bubble formation after a 48-h exposure by means of the BORA model, calibrated with data from previous air saturation dives. Compared to a 12-h exposure achieved in this study, the simulation of a 48-h exposure showed an increase in bubble formation. This increase was particularly significant for exposures to 50 msw. Previous studies [17], [18] have reported pain-only DCS, mainly confined to the diver’s knees, to be the predominant presentation when DCS occurs during saturation decompression. Medical management of this DCS type could probably be achieved without changing the decompression profile, using analgesics and additional periods of oxygen inhalation.

Most studies seem to indicate that there is a correspondence between detectable vascular bubbles and the incidence of DCS in saturation diving, but the number of type 1 DCS not accompanied by detectable bubbles seems to be higher than for subsaturation diving [7], [18], [19], [20]. Indeed, it is postulated that musculoskeletal manifestations of DCS are mainly caused by autochthonous gas bubbles and not systematically associated with detectable vascular bubbles [18]. As we have found only very low bubble grades (Spencer grades 0 or I) during decompression from exposures to 40 and 50 msw, we believe that DCS risk remains low. The simulations predict a higher DCS risk in subjects exposed to compressed air for 48 h compared to the present Nitrox schedules for 12 h (Table 1).

### Effects on Lung Mechanics

Two main mechanisms are recognized affecting pulmonary function of subjects exposed to increased ambient pressure. Firstly, due to the increase in gas density flow rates decrease as gas flow is changing from laminar to turbulent dependent of depth and ventilation requirements [21]. Static lung volumes, like vital capacity, are not affected by changes in ambient pressure [22]. The other mechanism affecting pulmonary function is hyperoxia. The decrease in VC is recognized as a major and early sign of POT that can be observed in the absence of clinical symptoms [23], [24]. Pulmonary gas exchange is also affected by hyperoxia as demonstrated by the reduced pulmonary diffusion capacity [25]. Restoration of pulmonary function is a slow process that can take several days or several weeks depending on the initial level of pulmonary alterations [8], [24].

**Figure 10:**
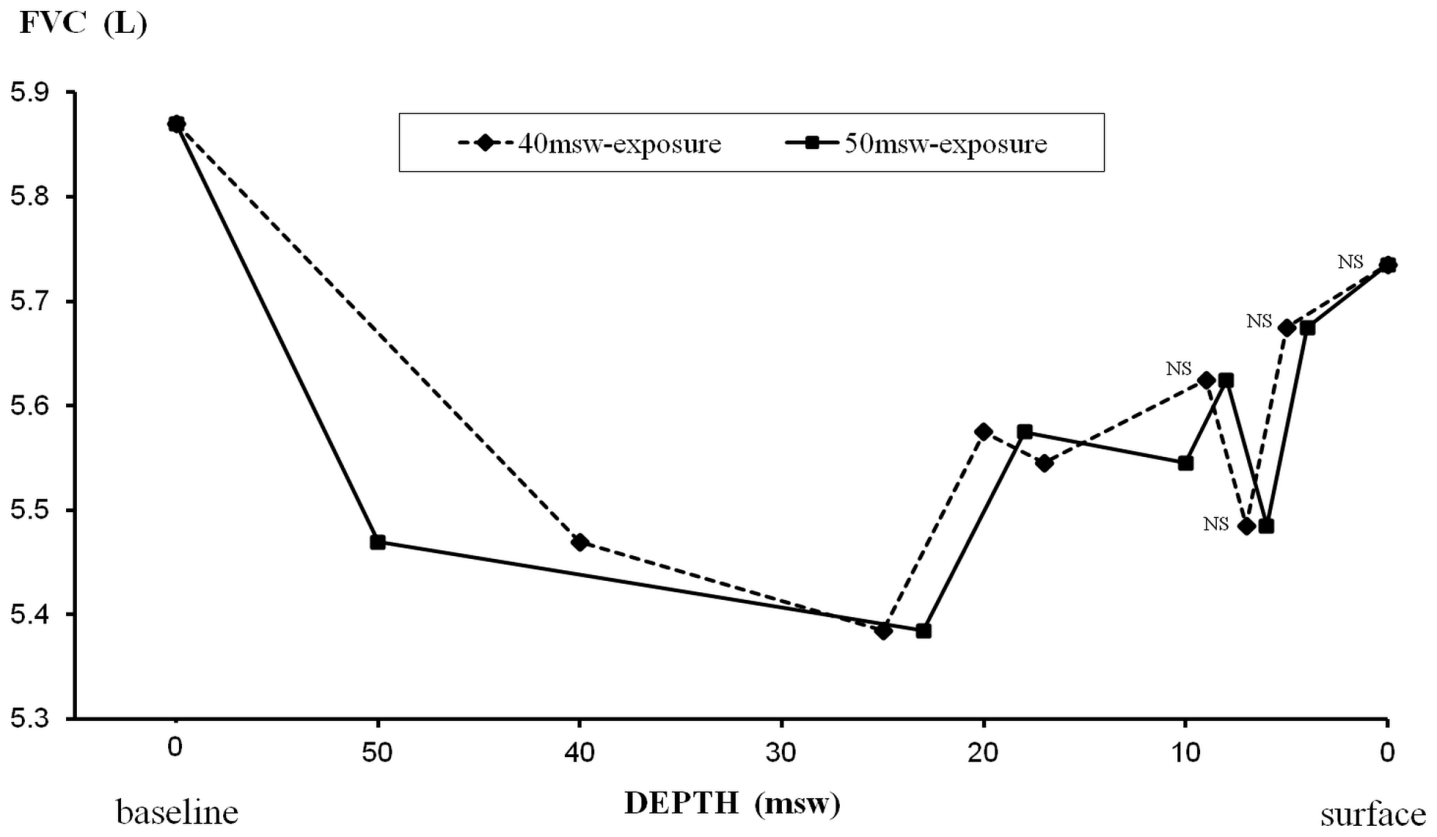
Changes in FVC during exposures to 40 msw (dashed line) and 50 msw (solid line). Statistically significant decrements from pre exposure values were found except for values indicated as non significant (NS). No statistical significant change compared to the pre exposure value observed during the 50 msw exposure.

In our study, the most pronounced effect of raised ambient pressure was observed on pulmonary flow rates during first 12 h at 40 and 50 msw, gradually subsiding during decompression. In contrast, the relative reduction of VC, suggesting POT, was at its peak during decompression, though a cumulative effect is obvious (Figure 10). From pre/post analysis, we found a decrease in FEF_25–75_ both for 40 and 50-msw exposures, while the decreases in FEV_1_ and FEV_1_/FVC were statistically significant only for 50-msw exposures (Table 2). The significant post-dive reduction in FEF_25–75_ suggests small airway dysfunction, possibly from an inflammatory process. This inflammation could be secondary to hyperoxia or result from the increase in mechanical shear forces due to the elevated gas density [26], [27]. The reduction in FVC, suggesting POT, was statistically significant only during exposures to 40 msw with a significant decrease of FVC values during decompression from 40 to 17 msw, with a maximum at 25 msw. We also found a slight decrease of less than 1% of DL_CO_ after the hyperbaric exposure. Since the cumulative dose of oxygen was quite similar in both groups (and even slightly higher for exposures to 50 msw), the difference in POT effect, statistically significant only for 40-msw exposures, can be considered paradoxical. Since substantial variability of POT among subjects was previously described [8], [24], it is conceivable that this difference may be related to inter-individual susceptibility in our study, with subjects more sensitive to the effects of hyperoxia in the group exposed to 40 msw. We believe that minor respiratory symptoms observed in both exposures from 30 to 20 msw were likely related to the combined effects of the gas density and oxygen toxicity, while late symptoms during O_2_ periods may be related to POT and inhalation of dry gas.

**Table 2:** Spirometry measurements pre and post exposure to 40 and 50 msw.

Exposure to 40 msw	PRE values	POST values	delta %
FVC (L)	5.7±1	5.8±0.7	1.8
TLC (L)	8.4±0.8	8.2±1	−2.4
RV/TLC (%)	30.8±4.9	29.5±4.8	−4.2
PEFR (L/s)	9.65±2	9.4±1.6	−2.6
FEV_1_ (L)	4.1±0.5	4±1.2	−2.4
FEV_1_/FVC (%)	73±10.5	71.3±13.1	−2.3
FEF_25–75_ (L/s)	3.8±2.75	3.1±3.15	−18.4*
DL_CO_ (mmol/min/kPa)	10.53±2.2	10.44±1.8	−0.9*
DL_CO_/VA (mmol/min/kPa/L)	1.58±0.3	1.5±0.3	−5.1*
**Exposure to 50 msw**	**PRE values**	**POST values**	**delta %**
FVC (L)	5.65±0.6	5.3±0.6	−6.2
TLC (L)	7.5±1.5	7.7±0.9	2.7
RV/TLC (%)	24.35±5.8	21.7±6.15	−10.9
PEFR (L/s)	10.3±2.8	9.6±2.2	−6.8
FEV_1_ (L)	4.4±0.5	4.2±0.6	−4.5*
FEV_1_/FVC (%)	77.8±6.9	76±8.7	−2.3*
FEF_25–75_ (L/s)	3.5±0.7	2.75±0.9	−21.4*
DL_CO_ (mmol/min/kPa)	12.5±1.8	12.3±2.6	−1.6
DL_CO_/VA (mmol/min/kPa/L)	1.7±0.2	1.6±0.15	−5.9

Statistically significant changes are indicated by an asterisk.

### Procedures for NSRS

During experiments of the Hydrolab Project from 1972 to 1975, 343 divers carried out air saturation at different depths up to 18 msw for periods ranging from 1 to 13 days [28]. Four decompression procedures have been published by NOAA in 1979: tables 12–10, 12–11, 12–12 and 12–13. Table 12–11 is the safest procedure, with only one reported case of joint DCS over 300 exposures in air atmosphere up to the maximum depth of 12.6 msw [28]. Greater depths of air saturation have been tested on a limited number of subjects [28], [29]. In a large experimental study, the US Navy Experimental Diving Unit evaluated accelerated decompression procedures using pure oxygen in a significant number of subjects i.e. 175 man-dives [6]. The incidence of DCS (17 incidents were observed) and circulating bubbles was monitored after ten alternative decompression schedules from Nitrox saturations between 40 and 60 feet (12–18 msw equivalent air depth). However, for deeper depths, the risk of severe POT prohibits the use of these procedures. Actually the submariners may be suffering symptoms of POT even before arrival of rescue, due to hyperoxia in the pressurized submarine. In this case, it is crucial to restrict additional hyperoxia during the succeeding decompression as this may worsen symptoms of POT. We did not observe any major reduction in pulmonary function, and we consider the use of these procedures acceptable for NSRS attendants - providing limited exposure time to maximum pressure. If we strictly apply the Repex method without exceeding the maximum cumulative doses given at 24 h, 48 h and 72 h, the maximum duration allowed time for attendants in air atmosphere is 2 h at 40 msw, but no exposure time at 50 msw would be allowed. However, the Repex guidelines [9] were developed for operational diving, and the procedures address the allowance needed for hyperbaric oxygen treatment in case of decompression sickness – conventionally a US Navy Treatment Table 6. Such a treatment table would add additionally 600 OTU. But in the context of NSRS, during decompression, Table 6 can not be performed. Therefore, we believe that an excess in OTU thresholds of about 1000 OTU for 24 h and 1500 OTU for 48 h could be tolerated. Based on the present decompression procedures for the 50 msw exposure, such an OTU allowance would permit a maximum exposure time (before decompression) of 12 h at 30 msw, 5 h at 40 msw and 1 h at 50 msw. Concerning the crew members from a disabled submarine, the results from AIRSAT 4 experiments indicated that the subjects should not significantly increase their pulmonary symptoms during decompression [8]. Clinical symptoms of POT could be improved by limiting O_2_ periods during decompression. Unless decompression rate is slowed further, such reduction of hyperoxia would be expected to increase the risk of DCS as predicted by our simulations of gas formation (Table 1).

In conclusion, the proposed procedure using slow decompression rates in air atmosphere and including periods of O_2_ breathing did not result in serious symptoms of decompression sickness or pulmonary oxygen toxicity. In scenarios where there are no operational constraints of decompression time, we believe that the procedure can be considered a first-line choice when the internal submarine pressure ranges 2.8–6 bar (18–50 msw). Further studies could be interesting to test in animal models emergency situations with rapid decompression and evaluate preventive measures to limit decompression stress or oxygen toxicity using specific biochemical markers.
